# Supplementary material for: A hybrid approach for intrusion detection in vehicular networks using feature selection and dimensionality reduction with optimized deep learning
Source: PLoS One. 2025 Feb 6;20(2):e0312752. doi: 10.1371/journal.pone.0312752 (PMC11801628; doi:10.1371/journal.pone.0312752)
Supplement: S1 Appendix — (PDF) [file pone.0312752.s001.pdf]

Appendix

Pseudocode: Hybrid Intrusion Detection System (Binary and Multilabel Classification.

Algorithm 1: Hybrid Intrusion Detection System for VANETs

```
Input: CICIDS2017 dataset, Labels (Benign, Attack Types)
Output: Trained model for intrusion detection (binary or multilabel)

Phase 1: Data Preprocessing
Load dataset, remove missing/invalid data, scale values
if Binary then
  | Group attacks into a single class (Malicious)
else
  | Retain each attack type as individual classes
end
Split dataset into 70% train, 30% test ensuring class balance

Phase 2: Feature Selection & Dimensionality Reduction
Apply CFS {95% or 90%} to remove highly correlated features
else Use PCA with 90% or 95% variance threshold for dimensionality reduction

Phase 3: Train Fully Connected Neural Network (FCNN)
Initialize FCNN with layers: Input (selected features), Hidden [64, 32, 16]
  neurons
if Binary then
  | Output: 2 neurons (Softmax)
  | Loss: Binary cross-entropy, Metrics: F1, Precision, Recall
else
  | Output: Number of attack classes (Softmax)
  | Loss: Categorical cross-entropy, Metrics: Macro-averaged F1, Precision,
  |   Recall
end
Compile FCNN with Adam optimizer (lr = 0.001), train for 40 epochs (early
  stopping)
Batch size: 32

Phase 4: Post-Training Quantization
Quantize model: Convert weights to 16-bit or 8-bit integers
Save the quantized model for deployment

Phase 5: Model Evaluation
if Binary then
  | Evaluate F1, Precision, Recall, Accuracy
else
  | Evaluate macro-averaged F1, Precision, Recall, Accuracy
end
Record model classification performance and model size before and after
  quantization
```

Feature Selection Results

Feature Counts for Different Selection Methods

Selection Method	Number of Features
Total Features	70
CFS95	47
CFS90	38

Total Features (70)

**Total Features**		
Destination Port	Flow IAT Min	Down/Up Ratio
Flow Duration	Fwd IAT Total	Average Packet Size
Total Fwd Packets	Fwd IAT Mean	Avg Fwd Segment Size
Total Backward Packets	Fwd IAT Std	Avg Bwd Segment Size
Total Length of Fwd Packets	Fwd IAT Max	Fwd Header Length.1
Total Length of Bwd Packets	Fwd IAT Min	Subflow Fwd Packets
Fwd Packet Length Max	Bwd IAT Total	Subflow Fwd Bytes
Fwd Packet Length Min	Bwd IAT Mean	Subflow Bwd Packets
Fwd Packet Length Mean	Bwd IAT Std	Subflow Bwd Bytes
Fwd Packet Length Std	Bwd IAT Max	Init_Win_bytes_forward
Bwd Packet Length Max	Bwd IAT Min	Init_Win_bytes_backward
Bwd Packet Length Min	Fwd PSH Flags	act_data_pkt_fwd
Bwd Packet Length Mean	Fwd URG Flags	min_seg_size_forward
Bwd Packet Length Std	Fwd Header Length	Active Mean
Flow Bytes/s	Bwd Header Length	Active Std
Flow Packets/s	Fwd Packets/s	Active Max
Flow IAT Mean	Bwd Packets/s	Active Min
Flow IAT Std	Min Packet Length	Idle Mean
Flow IAT Max	Max Packet Length	Idle Std
Flow IAT Min	Packet Length Mean	Idle Max
Fwd IAT Total	Packet Length Std	Idle Min
Fwd IAT Mean	Packet Length Variance	Label
Fwd IAT Std	FIN Flag Count	
Fwd IAT Max	SYN Flag Count	

Features Selected by CFS95 (47)

**CFS95 Features**		
Destination Port	Bwd IAT Total	PSH Flag Count
Flow Duration	Bwd IAT Mean	ACK Flag Count
Total Fwd Packets	Bwd IAT Std	URG Flag Count
Total Length of Fwd Packets	Bwd IAT Max	Down/Up Ratio
Fwd Packet Length Max	Bwd IAT Min	Init_Win_bytes_forward
Fwd Packet Length Min	Fwd PSH Flags	Init_Win_bytes_backward
Fwd Packet Length Mean	Fwd URG Flags	act_data_pkt_fwd
Bwd Packet Length Max	Fwd Header Length	min_seg_size_forward
Bwd Packet Length Min	Bwd Header Length	Active Mean
Flow Bytes/s	Bwd Packets/s	Active Std
Flow Packets/s	Min Packet Length	Active Max
Flow IAT Mean	Max Packet Length	Active Min
Flow IAT Std	Packet Length Mean	Idle Std
Flow IAT Max	Packet Length Variance	
Flow IAT Min	FIN Flag Count	
Fwd IAT Mean	RST Flag Count	

Features Selected by CFS90 (38)

**CFS90 Features**		
Destination Port	Bwd IAT Total	Init_Win_bytes_forward
Flow Duration	Bwd IAT Mean	Init_Win_bytes_backward
Total Fwd Packets	Bwd IAT Std	act_data_pkt_fwd
Total Length of Fwd Packets	Bwd IAT Max	min_seg_size_forward
Fwd Packet Length Max	Bwd IAT Min	Active Mean
Fwd Packet Length Min	Fwd PSH Flags	Active Std
Fwd Packet Length Mean	Fwd URG Flags	Active Max
Bwd Packet Length Max	Fwd Header Length	Active Min
Bwd Packet Length Min	Bwd Header Length	Idle Std
Flow Bytes/s	Bwd Packets/s	
Flow Packets/s	Min Packet Length	
Flow IAT Mean	Max Packet Length	
Flow IAT Std	Packet Length Mean	
